# Variability in baseline laboratory measurements of the Brazilian Longitudinal Study of Adult Health (ELSA-Brasil)

**DOI:** 10.1590/1414-431X20165381

**Published:** 2016-08-01

**Authors:** R. Ladwig, A. Vigo, L.M.G. Fedeli, L.E. Chambless, I. Bensenor, M.I. Schmidt, P.G. Vidigal, C.D. Castilhos, B.B. Duncan

**Affiliations:** 1 Programa de Pós Graduação em Epidemiologia, Faculdade de Medicina e Hospital de Clínicas de Porto Alegre, Universidade Federal do Rio Grande do Sul, Porto Alegre, RS, Brasil; 2 Laboratório Clínico, Hospital Universitário, Universidade de São Paulo, São Paulo, SP, Brasil; 3 Department of Biostatistics, University of North Carolina, Chapel Hill, NC, USA; 4 Departamento de Clínica Médica, Faculdade de Medicina, Universidade de São Paulo, São Paulo, SP, Brasil; 5 Departamento de Propedêutica Complementar, Faculdade de Medicina, Universidade Federal de Minas Gerais, Belo Horizonte, MG, Brasil

**Keywords:** Multi-center studies, Laboratory measurements, Quality control

## Abstract

Multi-center epidemiological studies must ascertain that their measurements are accurate and reliable. For laboratory measurements, reliability can be assessed through investigation of reproducibility of measurements in the same individual. In this paper, we present results from the quality control analysis of the baseline laboratory measurements from the ELSA-Brasil study. The study enrolled 15,105 civil servants at 6 research centers in 3 regions of Brazil between 2008–2010, with multiple biochemical analytes being measured at a central laboratory. Quality control was ascertained through standard laboratory evaluation of intra- and inter-assay variability and test-retest analysis in a subset of randomly chosen participants. An additional sample of urine or blood was collected from these participants, and these samples were handled in the same manner as the original ones, locally and at the central laboratory. Reliability was assessed with the intraclass correlation coefficient (ICC), estimated through a random effects model. Coefficients of variation (CV) and Bland-Altman plots were additionally used to assess measurement variability. Laboratory intra and inter-assay CVs varied from 0.86% to 7.77%. From test-retest analyses, the ICCs were high for the majority of the analytes. Notably lower ICCs were observed for serum sodium (ICC=0.50; 95%CI=0.31–0.65) and serum potassium (ICC=0.73; 95%CI=0.60–0.83), due to the small biological range of these analytes. The CVs ranged from 1 to 14%. The Bland-Altman plots confirmed these results. The quality control analyses showed that the collection, processing and measurement protocols utilized in the ELSA-Brasil produced reliable biochemical measurements.

## Introduction

The validity of inferences from clinical and epidemiological studies critically depends on the validity and reliability of measurements in the data collection process. Quality assurance and quality control measures are therefore needed throughout the stages of planning and data collection. For an individual patient in continued care, the use of quality specifications based on the intraperson biological variation of an analyte through a certain period of time is internationally accepted (1). For epidemiological studies, in which greater laboratory precision facilitates the detection of associations between variables, quality can be assessed through the agreement of repeated measurements from biological samples of a single participant visit (2).

Investigators in Brazil and in other low and middle income countries have increasingly been involved in multi-center studies with centralized laboratory analysis, but the necessary procedures for quality control have been infrequently reported. The ELSA-Brasil (Estudo Longitudinal de Saúde do Adulto - Brazilian Longitudinal Study of Adult Health) study utilized a central laboratory for most of its analyses, offering an opportunity to describe the methodology and results of the quality assessment of these measurements.

The resource of having a centralized laboratory in the ELSA-Brasil allowed each research center to have a team for the collection and processing of the biological material, which would be stored in cryotubes at -80°C for up to 30 days, and further transported to the central facility. The negative aspects of this strategy were the need for centralized training and certification of the teams from each center, and the increased risk of delay in the return of results to participants. The most important positive aspects were the facility for acquisition of the lab kits and the absence of inter-laboratory variability. The local processing of the samples decreased the volume of material transported, resulting in a reduction of costs.

The objective of this report is to present the results of quality control analyses of baseline laboratory measurements of the ELSA-Brasil.

## Material and Methods

### Data collection

The ELSA-Brasil study enrolled 15,105 participants in 6 field centers located in 3 different regions of the country from 2008 to 2010 (3). The study protocol was approved by ethics committees at each institution, and all participants gave their written informed consent. Participants underwent interviews, examinations and collection of blood and urine specimens, in approximately 6 h at the local research clinics.

As has been previously described in more detail (4), blood collection was performed in a fasting state and then, among those without a diagnosis of diabetes, 2 h after the ingestion of a 75-g oral glucose solution.

Blood was centrifuged within 30 min of collection, with aliquots then being separated in an ice bath into cryotubes previously labeled with bar codes, and stored in freezers at -80°C until transportation to the central laboratory on dry ice. Once samples were received, analysis for glucose (fasting and 2 h), glycated hemoglobin (HbA1c), creatinine, sodium, potassium, uric acid, aspartate transaminase (AST), alanine transaminase (ALT), gamma-glutamyl transferase, total cholesterol, HDL-cholesterol, LDL-cholesterol, triglycerides, thyroid stimulating hormone (TSH), insulin (fasting and 2 h) and ultrasensitive C-reactive protein, as well as serology for Chagas disease were performed.

Urine was collected over 12 h during the night, prior to the clinic visit, locally processed and stored for shipment in aliquots as described above. Determinations were performed centrally for sodium, potassium, calcium, creatinine and albumin.

Given the large number of participants, multi-center nature and diversity of measurements, the ELSA-Brasil required effective and efficient mechanisms of quality assurance and control for laboratory determinations. As previously reported (5), the main quality assurance activities were careful selection of research instruments, centralized training and certification, pretesting and pilot studies, and preparation of procedure manuals.

For laboratory measurements, inter-assay CVs were calculated using the results of internal controls, and intra-assay CVs using data from several pilot studies on both fresh and frozen samples.

In addition, we performed test-retest analyses of specific analytes in 10% of a randomly selected sample of study participants, with determination of intra- and inter-assay variability at the central laboratory. In selected participants, a single additional blood or urine sample was collected for duplication. Extra blood samples were collected at the end of the blood drawing process. These tubes ("blind replicates"), identified only by their bar code and thus, blind for determination, were handled identically to the original tubes and sent to the central laboratory at the same time as the original tubes.

Laboratory results were transmitted to the study's data center, where the original and quality control (QC) results were matched through the bar codes to individual study participants.

### Statistical analysis

Reliability was estimated through the calculation of intraclass correlation coefficient (ICC) (6), using a random effects model. A mixed model that also included a fixed order effect (QC sample minus original sample) was used for the four analytes for which the fixed effect was statistically significant at the 0.05 level. Confidence intervals were estimated by the 2.5 and 97.5 percentiles of the empirical distribution obtained with bootstrap sampling with 1000 repetitions.

We calculated the CV as the ratio of the standard deviation of measurement error to the mean of the analyzed variable.



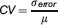



Bland-Altman plots were also produced to graphically explore the agreement between measurements (7).

Analyses were performed initially on all pairs. To minimize the effect of extreme outliers, we then reanalyzed the differences after removing pairs presenting a difference between measurements >3 standard deviations from the mean or for whom the pair's mean was >5 standard deviations from the overall sample mean. Analyses were performed using SAS software (USA), version 9.3 (8).

## Results

The inter- and intra-assay CVs for the analytes are reported in Table 1. This table also presents external, analytical CVs (CVa) specifications based on biological variation, for comparison. CVs obtained were generally quite small. Intra-assay CVs varied from 0.86% for HbA1C to 3.97% for urinary calcium; inter-assay CVs varied from 1.28% for sodium to 7.77% for insulin. Most CVs were lower than the CVa, with serum creatinine, serum sodium, and glycated hemoglobin being notable exceptions.

**Table:**
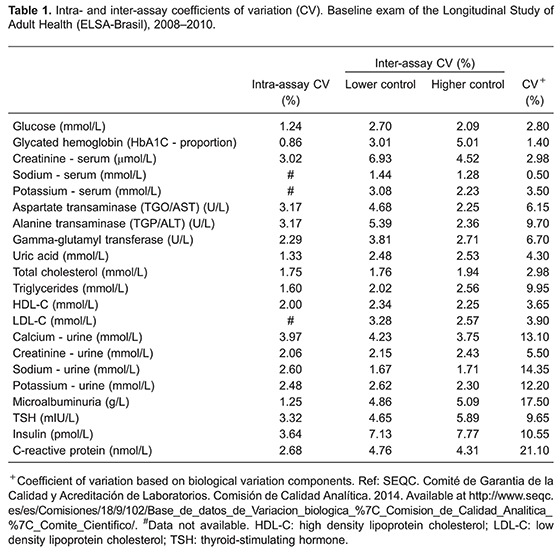

The number of blind replicate QC samples obtained varied from 72 to 94 across analytes. Fewer QC samples were available for analytes obtained 2 h after the glucose load, as participants with diabetes were excluded from this test.

Table 2 presents QC results for all blind replicate pairs for each analyte, and Table 3 shows results for pairs after the removal of outliers. The number of pairs removed varied from 0 to 4. All analytes had an ICC above 0.93 except for serum sodium (0.50) and potassium (0.73) (Table 2). The removal of pairs with outliers (Table 3) generally produced a small improvement in ICCs values, except for serum sodium, which increased from 0.50 to 0.61.

**Table:**
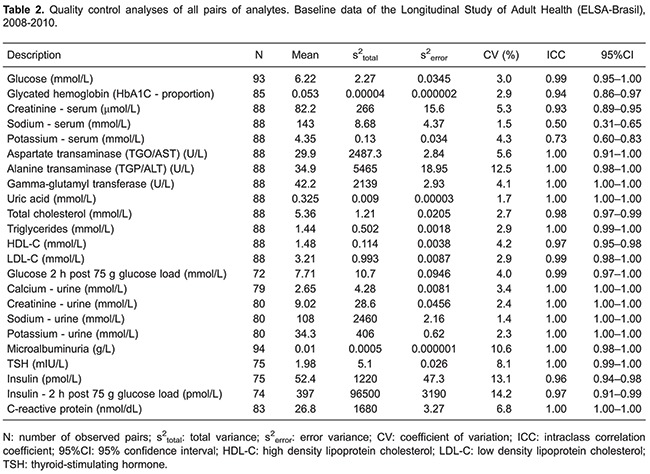

**Table:**
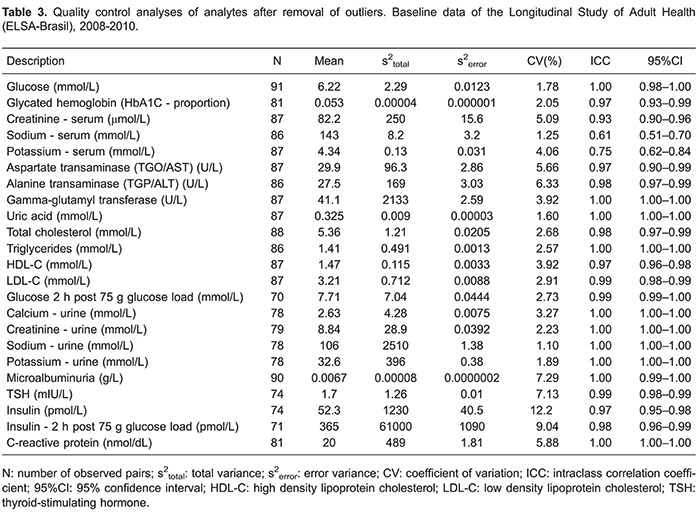

**Figure 1:**
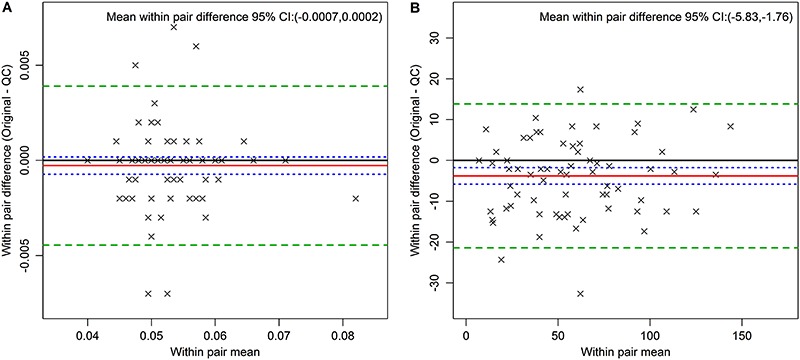
Bland Altman plots for glycated hemoglobin. Proportion of total hemoglobin (all pairs, *Panel A*) and insulin in pmol/L (all pairs, *Panel B*). The red line indicates the mean within-pair difference found between the original and the quality control (QC) measures. The green dashed lines indicate 2 standard deviations above and below the mean, and the blue dotted line the 95%CI for the mean of the within-pair difference.

Figure 1 presents Bland-Altman plots of the pair differences against average pair values for selected analytes. Panel A shows data for glycated hemoglobin and Panel B for fasting insulin, the latter demonstrating a small systematic difference between initial analyte and the QC sample (-3.79; 95%CI: -5.83 to -1.76). Other similarly small differences (Supplementary Figures) were seen between pairs for serum creatinine (2.34; 95%CI=1.19–3.49), serum potassium (0.16; 95%CI=0.12–0.20), and TSH (0.06; 95%CI=0.01–0.11). Although both glycated hemoglobin and insulin had outliers, their removal had only a small effect on CV and ICC values.

**Figure 2:**
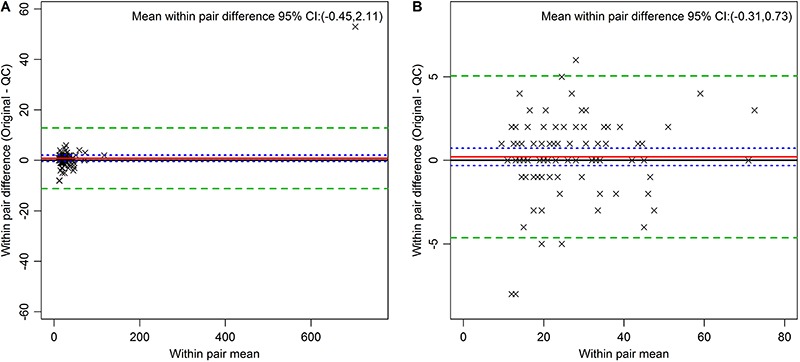
Bland Altman plots for alanine transaminase in U/L (all pairs, *Panel A*; after exclusion of outliers, *Panel B*). The red line indicates the mean within pair difference found between the original and the quality control (QC) measures. The green dashed lines indicate 2 standard deviations above and below this mean, and the blue dotted line the 95%CI for the mean of the within pair difference.

In order to visualize the pairs and the influence of outliers, Figure 2 shows results for alanine transaminase, prior to (Panel A) and after exclusion of outliers (Panel B). As can be seen in Panel A, a single extreme outlier, probably due to liver disease, makes QC analysis difficult. In fact, as can be seen when comparing results for this analyte in Tables 2 and 3, after outlier exclusions the CV decreased from 12.5 to 6.33%. The ICCs, prior to and after this exclusion were high with laboratory variability accounting for less than 2% of the overall variance.

**Figure 3:**
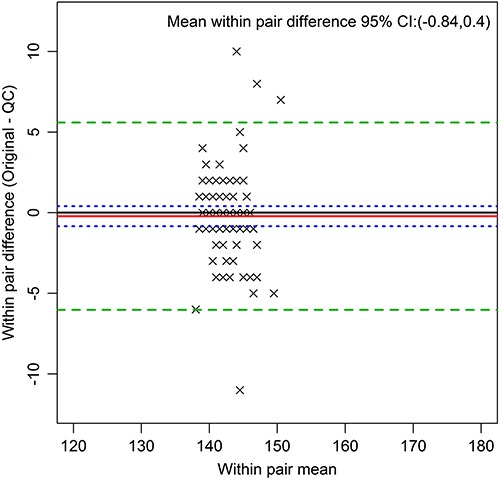
Bland Altman plot of serum sodium in mmol/L (all pairs), an example of an analyte with a narrow biological range. The red line indicates the mean within pair difference found between the original and the quality control (QC) measures, the green dashed lines indicate 2 standard deviations above and below this mean, and the blue dotted line the 95%CI for the mean of the within pair difference.

Figure 3 presents the Bland-Altman plot for serum sodium, demonstrating the narrow biological range of this analyte.

## Discussion

This study demonstrates the excellent quality of the sample collection and processing, and laboratory measurement of biochemical analytes of the ELSA-Brasil study, in a sample of free-living Brazilian adults. Laboratory intra- and inter-assay variability was almost always below recommended international standards. Between-person variability accounted for more than 90% of the total variability with few exceptions, and was usually greater than 98%. For epidemiological studies involving associations between exposures and diseases, the most inclusive and relevant of the QC measures is the ICC. The ICC, also called the reliability coefficient, can be interpreted as a ratio of the between-individual variance to the total variance. The existence of noise (underlying within-person variability) as a percentage of the signal (true value of exposure, in the case the laboratory values) lowers the ICC values. Therefore, when testing epidemiological associations, the greater the noise the more difficult it is to detect real associations at a level of statistical significance. One important, and partially controllable source of this variability is the intraindividual variation, sometimes called "measurement error". The overall reliability coefficient evaluates the fraction of the population variability of a given variable that are not due to measurement errors (intra-individual variability). This measurement error could originate from biological variation or from either pre-analytical or analytical sources. Here, with samples taken from a single participant visit, the biological variation was not evaluated. Pre-analytical errors include those related to variability in collection (e.g., hemolysis), processing (e.g., delay in chilling and centrifugation leading to metabolism of glucose in the collection tube), storage (e.g., inadequate temperature maintenance) and transportation (e.g., premature thawing). Also to be considered is the possible mixing of tubes among participants during the collection processes. In this study, none of the outliers were clustered to a particular individual participant, indicating that the possibility of serious errors resulting from switching or erroneously labeling collection tubes or aliquots, or assigning laboratory results to the wrong participant, seem unlikely.

Analytical errors include laboratory determination errors (e.g., reagent variability between kits). The intra- and inter-assay CVs available from the ELSA central laboratory indicate very small error in this part of the measurement process, in general. With few exceptions, VCs of measured analytes were lower than the analytical quality specifications based on components of biological variation (9). Of note, some analytes, such as insulin, are typically measured with greater variability.

Thus, the bulk of the variability in reported measurements seems likely to be originated from a combination of smaller problems in analyte collection, processing and transportation, coupled with the specific analyte susceptibility to each of these problems.

The markedly lower ICCs of the analytes serum sodium and potassium highlight an interesting phenomenon. As the CVs for these analytes were quite high, the low ICCs can be attributed to their low between-person variability, as illustrated by the narrow distribution of the values in the Bland-Altman plots for serum sodium (Figure 3) and potassium (Supplementary Figures). The ranges of these analytes, vital to body function, are tightly controlled within narrow limits. Despite this, their ICCs indicated that their relatively high measurement error would make it more difficult to find statistically significant associations involving either of them in the ELSA sample.

The CV, popular in reports of measurement error, is of utility in determining the extent to which the analyte value of a given participant will vary, in relation to the analyte's mean value, from one measurement to the next - a relevant issue in the determination of the analyte's clinical utility. However, as CV evaluation doesn't take other variabilities into consideration, it has a lesser role in evaluating the quality of measurements in epidemiologic studies of associations. In this study, the blind replicate CVs were higher than the intra- and inter-assay CVs, which was caused by the inclusion of not only laboratory measurement variability, but also pre-analytical sources of error usually present in multi-center studies with centralized measurements.

The Bland-Altman plots provide a visual analysis of these QC results. One can see potentially systematic differences among replicates, such as that found for fasting insulin (Figure 2). This difference, when present, was always small, and most likely due either to the fact that the QC sample was always drawn last, or to chance, given the multiple comparisons performed. The plots show no evidence of increased or decreased variability as a function of the analyte average value, which might be present, for example, if inadvertent thawing had led to loss of analytes. The +/- 2 standard deviations boundaries were relatively close to the means, once again indicating a small measurement error.

The decision to report results with and without outliers is not always easy. For example, it seems logical to use the ICC and CV obtained for alanine transaminase after exclusion of the outlier seen in the Bland-Altman plot, given that the mean of the outlier pair was more than 30 times the mean of the remaining pairs, leading to a potentially large weighting of this single value. The outlier value was apparently obtained from a participant with liver disease and, given the large difference within the pair (>50 U/L), likely outside the range of precision of the measurement technique. For precision evaluation in an epidemiological study in which the overwhelming majority of participants have values within or near the normal range, inclusion of such outliers makes interpretation of the findings difficult. As can be seen when comparing the Bland-Altman plots before and after the outlier exclusion, it obscures the analysis of measurement error of values within the relevant range. In contrast, outliers in other analytes, both for average values and those for differences between the QC sample and its pair, were close to the exclusion boundaries. As such, these values should probably be considered measurements within the relevant range of study, and their differences should be included in our final estimates of measurement variability.

Two limitations of our analysis merit mention. First, as QC samples were collected, processed, and shipped simultaneously with their corresponding pair samples, some of the measurement variability occurring during these steps could have been underestimated. Additionally, an important aspect of variability - temporal biological variability - was not assessed, as participants were not recalled to repeat measurements on another day. Thus, for a thorough evaluation of the measurement reliability for epidemiological studies, consideration of this within-participant day-to-day variability would require incorporation of data from other studies.

In conclusion, these analyses of measurement variability are important for QC documentation in multi-center studies during which strict QC measures are necessary to guarantee accurate results. The biochemical analytes of the ELSA-Brasil here reported were measured with high reliability. Based on this, they should serve well as exposure variables and co-variables for most studies of associations, especially given the large sample size of the cohort.

## Supplementary material

Click here to view [pdf].
